# A Synopsis of Serum Biomarkers in Cutaneous Melanoma Patients

**DOI:** 10.1155/2012/260643

**Published:** 2012-01-12

**Authors:** Pierre Vereecken, Frank Cornelis, Nicolas Van Baren, Valérie Vandersleyen, Jean-François Baurain

**Affiliations:** ^1^Department of Dermatology, Centre Hospitalier Valida and Cliniques Universitaires Saint-Luc, B-1082 Brussels, Belgium; ^2^Department of Medical Oncology, Cliniques Universitaires Saint-Luc, Brussels, Belgium; ^3^Brussels Branch, Ludwig Institute for Cancer Research, Brussels, Belgium

## Abstract

Many serum biomarkers have been evaluated in melanoma but their clinical significance remains a matter of debate. In this paper, a review of the serum biomarkers for melanoma will be detailed and will be discussed from the point of view of their practical usefulness. The expression of biomarkers can be detected intracellularly or on the cell membrane of melanoma cells or noncancer cells in association with the melanoma. Some of these molecules can then be released extracellularly and be found in body fluids such as the serum. Actually, with the emergence of new targeted therapies for cancer and the increasing range of therapeutic options, the challenge for the clinician is to assess the unique risk/response ratio and the prognosis for each patient. New serum biomarkers of melanoma progression and metastatic disease are still awaited in order to provide efficient rationale for followup and treatment choices. LDH as well as S100B levels have been correlated with poor prognosis in AJCC stage III/IV melanoma patients. However, the poor sensitivity and specificity of those markers and many other molecules are serious limitations for their routine use in both early (AJCC stage I and II) and advanced stages of melanoma (AJCC stage III and IV). Microarray technology and proteomic research will surely provide new candidates in the near future allowing more accurate definition of the individual prognosis and prediction of the therapeutic outcome and select patients for early adjuvant strategies.

## 1. Introduction

The incidence of cutaneous malignant melanoma (CMM) is still increasing in the western world despite early detection and prevention campaigns. Patients are mostly young and late diagnosis, which means thicker tumors (thicker than 1 mm, or Breslow index ≥1 mm: the Breslow index is the measurement in mm of the vertical thickness of the primary tumor) and/or involvement of regional lymph nodes, causes a greater risk of developing a disseminated disease. CMMs usually progress from an in situ proliferation to a radial growth pattern, and then to a vertical growth phase. This vertical growth phase represents a key event for the cell spread, since it allows the cells to migrate deeply in the dermis, in the lymphatics, and the bloodstream.

In the 7th revision of the American Joint Committee on Cancer (AJCC) for melanoma staging and classification (2009), patients can be divided in four stages, from stage I and II (local disease) to stage III (locoregional disease) and stage IV (metastatic disease). In this classification, the only marker which has been incorporated for clinical use is lactate dehydrogenase (LDH) since elevated serum LDH has been shown in multivariate analysis to be an independent and highly significant predictor of survival even after accounting for site and number of metastases. 

Surgery remains the mainstay of the melanoma treatment. Actually, the major concern after the diagnosis by primary surgery or primary excision is to know whether this cancer has already metastasized or not. Indeed many arguments emphasize that early detection of melanoma metastasis could improve the prognosis of patients, at least for a part of them.

High-risk melanoma patients can be defined by a 50% risk of relapse despite initial optimal surgical treatment. This group of patients should be carefully followed and if possible treated by efficient adjuvant therapeutic strategies. Interferon-*α* and more recently ipilimumab have been proposed as adjuvant treatments but their effect on survival is still a matter of debate. To date no predictive factor of response has been described.

**Figure 1:**
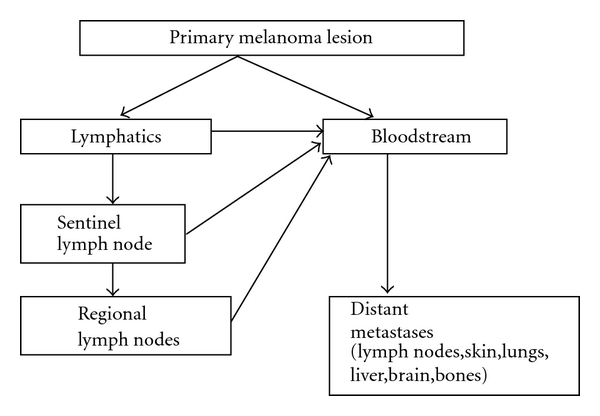
The process of metastasis is the consequence of migration of melanoma cells from the primary lesion, to locoregional and distant body sites via the lymphatic circulation and the bloodstream. Sentinel lymph node is the first draining lymph node in which melanoma cells may find a suitable environment and survive, leading to micro- or macrometastasis. If the cells spread hematogenously, distant metastasis will appear.

The process of metastasis involves the spread of neoplastic cells to locoregional or distant body sites via lymphatic vessels and/or bloodstream. In the case of melanoma, circulating cells may find a suitable microenvironment in the first draining lymph node, known as the sentinel lymph node, in other lymphnodes or in distant organs, leading to secondary tumor growth (Figure 1). Melanoma may spread to almost all organs, with predilection for lymph nodes, liver, lungs, brain, and bones. Understanding the biology and the mechanism of metastasis provides new molecular targets and may help us to discover new biomarkers.

When metastatic disease is confirmed late and surgery can no longer be chosen, therapeutic options are limited and give disappointingly low responses. These options include specific or nonspecific immunotherapy, chemotherapy, radiotherapy, radiosurgery, radiofrequency ablation.

## 2. Towards the Definition of a Biomarker in Cutaneous Malignant Melanoma?

Biomarkers can be divided into diagnostic markers for screening and prognostic markers, which can be used once the cancer has been diagnosed and predictive markers, which should predict the likely response to a treatment.

Cancer biomarkers include molecular tools such as proteins, peptides, DNA, mRNA, or processes which can be measured in a given cancer, with specific quantitative and qualitative tools. These markers can be found in tissues, cells, and/or body fluids. In addition viable melanoma cells can also be found in the peripheral blood of melanoma patients. The discussion will be limited here to serum biomarkers in melanoma patients.

The ideal serum biomarker should be a molecule detection which in the blood allows diagnosis of a growing tumor in a patient. The biomarker must exhibit sufficient sensitivity and specificity in order to minimize false negative and false positive results. The sensitivity refers to the proportion of patients with a confirmed disease who will have a positive test for a biomarker, while the specificity can be defined by the proportion of healthy individuals with a negative test.

Previous studies have shown that many molecules which may be involved in oncogenesis and cancer spread can be found in the serum of cancer patients in particular melanoma patients, but their sensibility and/or specificity is still questionable. These molecules can be produced and secreted or shed into the bloodstream directly by melanoma cells or indirectly through destruction of melanoma cells by chemotherapy, immunotherapy, or combined therapy. Biomarker discovery is a complex research process which involves scientific collaboration and data share. Early approaches rested on clinical and pathological findings, as illustrated by the CEA and the PSA stories, but now emerging technologies such as genomics and proteomics have influenced and changed the paradigm.

At this moment no ideal biomarker exists in the melanoma field, and additional markers (combined markers) provide probably more useful information as shown in some reports. Routine use of tumor markers is an important issue because it would allow early detection and definition of therapeutic strategy.

In addition, melanoma biomarker research is an open field for the understanding of molecular events in melanoma progression and should provide new molecular targets for therapeutic intervention.

Hereafter we detail the most important serum molecules which have been described as biomarker for CMM.

### 2.1. Common CMM Biomarkers

#### 2.1.1. Lactate Dehydrogenase (LDH)

As already discussed above, this enzyme has been considered as the main serum parameter in metastatic melanoma patients and identified as a “good” biomarker in metastatic patients. Many studies have presented LDH as the most predictive independent factor.

This led to a stratification in the American Joint Committee of Cancer staging system. Metastatic melanoma patients with high LDH levels are designated as M1c whatever the site of metastases.

However, Hamberg et al. [1] recently indicated that in a series of 53 AJCC stage IV melanoma patients only 38% had high levels of LDH, suggesting that elevated LDH is not the ideal marker for this condition. Moreover, in a multivariate analysis of 64 AJCC stage IV melanoma patients, Hauschild et al. [2] failed to demonstrate the independent prognostic value of LDH. It should be also remembered that LDH dosage can be falsely positive due to haemolysis and other factors among them hepatitis. Others markers are thus needed.

#### 2.1.2. C-Reactive Protein (CRP)

CRP is a nonspecific inflammatory parameter which might have a role in the detection of melanoma progression. This protein is produced by hepatocytes as a nonspecific acute phase response of inflammation processes.

High serum CRP levels have been linked to poor prognosis in various neoplasia. In a recent report, Deichmann et al. [3–5] analyzed the prognostic significance of C-reactive protein (CRP) compared to LDH in AJCC stage IV melanoma patients. With a cut-off point of 3 mg/dL, serum analysis discriminated between stage IV and nonstage IV melanoma patients, with a sensitivity of 76.9% and a specificity of 90.4%. In another prospective study of 67 patients, Deichmann considered that CRP alone was the most relevant prognostic parameter [3]. These results are debated. 

#### 2.1.3. S100-*β* Protein (S100B)

S100B protein is a 21-kd dimeric protein, consisting of two subunits *β*. This protein is a member of a 19 proteins family and was first isolated from bovine brain in the mid sixties. S100B protein is expressed by glial cells and melanocytes and has been shown to be produced by brain tumors and melanoma.

Roles of S100B are probably multiple and underestimated. It can interact with the p53 tumor suppressor gene in a calcium-dependent manner.

The serum S100B level is linked to the tumor burden and reflects clinical stage and tumor progression as reported by some.

**Figure 2:**
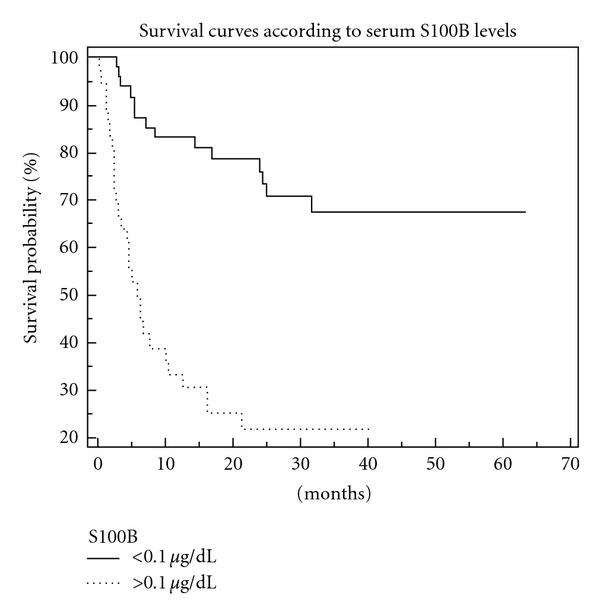
Survival curves according to serum S100B levels: a S100B > 0.1 *μ*g/dL is a bad prognosis factor (personal data).

There is increasing evidence that time-dependent evaluation of serial blood measurements of S100B is useful in order to follow melanoma patients (Figure 2); many reports have shown that S100B levels are correlated with clinical stage (the higher the level, the more advanced the stage) and could be used to monitor the effectiveness of antitumoral treatment whatever the type of the treatment (surgical, chemotherapy, immunotherapy). Retsas et al. [6] have even suggested the use of S100B instead of LDH in the AJCC staging system while other authors consider that S100B does not have any added value when comparing its sensitivity and specificity to CRP and LDH.

 S100B has probably become the most useful tumor marker in clinical practice but seems limited to advanced stage III and stage IV melanoma patients: in stages I and II S100B does not provide independent prognostic information. Currently S100B is not used routinely despite a high predictive value for the recurrences in this group of patients.

Moreover one should remember that S100B is not melanoma specific and that its serum level can be elevated in healthy subjects, nonmelanoma skin cancer patients, in neurological disorders, in AIDS, in central nervous system tumours, and even in various gastrointestinal cancers.

### 2.2. Novel Molecules Which Can Be Considered as Possible Biomarkers in CMM

#### 2.2.1. Melanoma Inhibitory Activity (MIA)

MIA is a 12 kDa soluble protein, the role of which has been characterized as an autocrine cell growth inhibitor. It can be expressed by melanoma cells as well as chondrocytes. The roles of this protein are multiple as the molecule may modulate cell growth and cell adhesion. MIA has been shown to be elevated in the serum of relapsing cases (Elisa test) and has been described as a useful marker to monitor melanoma patients after surgery. Some authors considered that sensitivity of both molecules MIA and S100B is equal. For some authors neither MIA nor S100B is superior to LDH and CRP on multiple logistic regression analysis. In children and pregnant women (after week 38), MIA is increased and MIA serum measurements should be thus avoided in these two groups [7].

#### 2.2.2. Galectin-3

Galectin-3 is the member of the family of lectins which can bind to *β*-galactosides. Many members of the galectin family are differentially expressed in cancer. Gal-3 is a molecule which contains an NH2-terminal domain, a COOH-terminal domain, and a collagen-like long sequence. In melanoma, Gal-3 has been shown to be overexpressed in malignant melanocytic lesions and also released in serum of melanoma patients by both melanoma cells and inflammatory cells. Gal-3 has been shown to play important roles in cell proliferation, cell differentiation, cell adhesion, cell migration, angiogenesis, and metastasis. Thus, Gal-3 deserves close attention, and clarifying the role of extracellular Gal-3 should help us to understand the significance of high serum levels of this molecule in advanced melanoma patients.

#### 2.2.3. Melanoma-Associated Antigens and Circulating Melanoma Cells

Malignant transformation of melanocytes causes changes in gene expression. This leads to the expression of molecules—so called melanoma associated antigens—which are more or less specifically associated with the malignant phenotype (Table 1). Sometimes these MAA can also be expressed in normal melanocytes but then can be observed in sequestered sites. These MAAs play important roles in triggering the antimelanoma immune response. These antigens have mostly been identified by immunological approaches, including in vitro and vivo reactions, and by serological tests. These antigens can be defined by their ability to interact with T or Bcells, and peptides derived from these antigens have been used to induce or sustain a specific antimelanoma immunological response. Mage-1 was the first MAA identified by a genetic approach in the Ludwig Institute for Cancer Research, Brussels, Belgium and this belongs to a broad family of at least 12 antigens differentially expressed by benign and malignant melanoma cells. Immune responses to these genes can be used as markers of disease and/or the existence of immune competence.

Polymerase chain reaction technique (PCR) is a technique which allows the detection of 1 tumor cell among 10^7^-10^8^ cells which is much more precise than light microscopy with a limit of detection of 1/100 –1/1000. PCR-based techniques rest on an exponential amplification of specific DNA or RNA molecules. With this technique, the identification of tumor-specific or tumor-associated genes leads to specific detection of tumor cells.

In RT-PCR serum analysis, RNA of the sample is first extracted and reversely transcribed into cDNA (reverse transcription). A gene of interest is then amplified thanks to specific primers, and isolated on agarose gel, or hybridized after southern blotting. Sequencing of the product of PCR can be carried out in order to compare it with the gene of interest.

Serum tyrosinase activity or positive tyrosinase RT-PCR in melanoma patients has been shown to be correlated with higher risk of relapse, but only 55% of these patients will experience a clinical relapse [8]. Moreover the specificity of this technique has yet to be optimized [9]. When combined with a S-100 assay, Domingo-Domenech showed that tyrosinase RT-PCR adds valuable prognostic information in patients with S-100 < 0.15 *μ*g/l, even if this team showed that S-100 had a higher predictive value.

Curry et al. [10] have suggested that RT-PCR detection of tyrosinase and MART-1 (Melanoma Antigen Recognized by T cells-1) positive circulating melanoma cells can be useful to determine a subgroup of patients with increased risk of metastasis.

#### 2.2.4. Melanin-Related Metabolites

5-S-cysteinyldopa is a precursor of phaeomelanin and is produced by both melanocytes and melanoma cells, as the product of the binding of a highly reactive molecule, dopaquinone, to cysteine. 5SCD has been shown to be detectable in the serum and in urine of melanoma patients and to correlate with disease progression. In progressive patients it has been previously published that 5SCD levels increased significantly earlier than clinical signs. A comparative report has stated that together with LDH and S100B, 5SCD was an interesting biomarker even if the authors of this report concluded that S100B could be regarded as the most sensitive of the three markers. Because of the effect of UV exposure on melanin pigment pathways the use of this 5SCD as a biomarker may be limited in Caucasians, while its use in Japan is more extensive. Moreover, patients with amelanotic metastasis usually do not have increased serum levels of 5SCD.

3,4-dihydroxyphenylalanine (L-dopa) is the first metabolite involved in melanogenesis and its plasma levels have been correlated with melanoma progression and tumor burden, as well as the plasma L-dopa/L-Tyrosine ratio which represents an index of tyrosinase and tyrosine hydroxylase activity. Stoitchkov et al. have shown that this latter ratio has a predictive value, especially in stage III patients, and advocated the simultaneous use of several biomarkers.

#### 2.2.5. Matrix Metalloproteinases (MMPs)

MMPs are a family of 24 structurally related endopeptidases. Theses zinc-dependent enzymes are defined by their own substrates and can lyse components of the ECM (for instance type IV collagen, which is a major component of basement membrane by gelatinases such as MMP-2 and MMP-9) and play a role in angiogenesis and turnover of the ECM. MMP may also cleave other molecules such as other proteinases, proteinase inhibitors, growth factors, adhesion molecules and in consequence modulate the inflammatory reaction, growth processes, tumor invasion, and metastasis.

A balance between MMP and tissue inhibitors metalloproteinases (TIMP) can be broken by an upregulation of MMPs or a downregulation of TIMPs as this can be shown in the acquisition of a malignant phenotype.

Another important role, angiogenesis, has been attributed to MMPs, and this could afford a possible therapeutic target. Batimastat (BB-94, a synthetic broad spectrum metalloproteinase inhibitor) for instance has been shown to inhibit angiogenesis of liver metastases in mouse models.

MMP expression has been reported during melanoma progression, and high serum levels of MMPs, namely, MMP-1 and MMP-3, have been correlated with poor survival.

#### 2.2.6. Cytokines, Chemokines, and Their Receptors

Chemokines are small signalling polypeptides that can bind to and activate G protein-coupled receptors, a family of seven transmembrane molecules. Multiple roles have been attributed to chemokines, and these molecules are implicated in the transformation and metastasis processes. Pattern expression of chemokines and their receptors could explain organ-specific metastasis.

Melanoma cells have been shown to express the chemokine CXCL8, also known as interleukin-8 (IL-8), and a report has established that high serum levels of IL-8 are associated with tumor burden and poor prognosis. This might be an interesting approach since in vivo studies have already demonstrated that anti-IL8 humanized antibodies are able to decrease tumor growth and angiogenesis.

Very recently an American team investigated 29 cytokines simultaneously (chemokines, angiogenic factors, growth factors, soluble receptors) with a high-throughput multiplex immunobead assay technology (Luminex Corp., Austin, Tex, USA) in the sera of 179 patients with high-risk melanoma and 378 healthy individuals. They were able to define a specific serum cytokine profile in the patients compared to healthy individuals—higher serum concentrations of interleukin (IL)-1alpha, IL-1beta, IL-6, IL-8, IL-12p40, IL-13, granulocyte colony-stimulating factor, monocyte chemoattractant protein 1 (MCP-1), macrophage inflammatory protein (MIP)-1alpha, MIP-1beta, IFN-alpha, tumor necrosis factor (TNF)-alpha, epidermal growth factor, vascular endothelial growth factor (VEGF), and TNF receptor II. Moreover they showed that IFN-alpha2b therapy resulted in a significant decrease of serum levels of immunosuppressive and tumor angiogenic/growth stimulatory factors and increased levels of antiangiogenic IFN-gamma inducible protein 10 (IP-10) and IFN-alpha. Finally they established a predictive value to the pretreatment levels of proinflammatory cytokines IL-1beta, IL-1alpha, IL-6, TNF-alpha, and chemokines MIP-1alpha and MIP-1beta which were found to be significantly higher in the serum of patients with longer RFS values [12]. Both IL-10 and soluble IL-2 receptors have been correlated to poor outcome [13, 14].

#### 2.2.7. Growth Factors and Angiogenesis Factor

**Vascular Endothelial Growth Factor (VEGF)**

Angiogenesis is an important step in tumor growth since it allows the delivery of oxygen and substrates. This process is the result of complex interactions between proangiogenic and antiangiogenic factors released by either tumor cells, native endothelial, epithelial, mesothelial cells and leucocytes. VEGF has been described as a potent mitogen of endothelial cells and a chemotactic factor also for monocytes and Tumor-Associated Macrophages (TAM's) and plays a key regulatory role during neoangiogenesis. Moreover, this growth factor is a vasopermeability stimulant and was formerly known as the Vascular Permeability Factor (VPF). Its expression has been correlated with tumor progression and prognosis and can be increased by hypoxic conditions.

Various VEGFs have been discovered and referred as VEGF-2, VEGF-3,…. Very recently, a tem has reported lower serum of VEGF-C levels in metastatic patients with skin/subcutaneous metastasis compared to metastatic patients with other distant sites [15]. In another study, VEGF serum level was not considered as in independent prognostic factor in multivariate analysis [16].

Very recently, a team has reported lower serum VEGF-C levels in metastatic patients with skin/subcutaneous metastasis compared to metastatic patients with other distant sites.

### 2.3. Others: Cell Surface and Adhesion Molecules

#### 2.3.1. Integrins

Integrins are cell components which assure adhesion to the other cells, the ECM, or to other proteins, as serum proteins. Other important roles can be played by integrins as mediation of information between extra- and intracellular space, angiogenesis.

Integrins are heterodimeric cell adhesion receptors composed of two subunits, *α* and *β*. On the basis of their common subunit, integrin heterodimers can be subdivided into *α*v, *β*1 and *β*2 integrins. The main integrins involved in melanoma progression include *α*v*β*3 (receptor for vitronectin and fibronectin), *α*2*β*1 (collagen), *α*4*β*1 (fibronectin), and *α*6*β*1 (laminin).

Some reports have shown that increased serum levels of *β* integrins have been associated with shorter survival. Clinical impact of this has yet to be defined.

#### 2.3.2. CD44

CD44 is a cell surface transmembrane glycoprotein, originally described as a homing receptor for lymphocytes. In the literature, this protein is described to play a role in tumor invasion and metastatic process. Multiple isoforms of CD44 are generated by alternative splicing of transcripts of its gene. CD44 is an important cell surface receptor for hyaluronan, and its downregulation 5CD44H, loss of CD44v3 expression has been correlated with poor outcome by some but not confirmed. Moreover serum level studies have also been conducted, which did not show any significance in defining the prognosis of melanoma patients.

#### 2.3.3. ICAM-1

ICAM-1 is an intercellular adhesion molecule which can be found in the cell membranes of leukocytes and endothelial cells. ICAM-1 is a ligand for LFA-1 (lymphocytes function-associated antigen-1, integrin family) of Tcells, Bcells, macrophages, and neutrophils. Migration of leukocytes is facilitated by ICAM-1/LFA-1 binding. Comparative measurements of serum levels of ICAM-1 and 5-S-CD have concluded that the level of 5-S-CD is a better marker for disease progression in melanotic melanoma, and another study showed that sICAM-1 was increased in metastatic patients but without independent prognostic value in multivariate analysis [17].

## 3. Discussion

Cancer is a major cause of morbidity and mortality in our society. It has exacted a tremendous price and has had many devastating effects. In particular, the rising incidence of cutaneous melanoma in western population is a major health problem.

Melanoma growth and progression are well defined in their clinical and histopathological patterns. The prognosis of CMM is strongly related to the stage at which it is diagnosed; with early diagnosis, a high proportion of lesions present a good prognosis.

If diagnosed late, melanoma can be a very aggressive malignancy. Moreover, melanoma may sometimes exhibit unpredictable clinical behaviour: the thickness of the primary lesion (Breslow index) is the most important prognostic factor but patients with thick melanoma (Breslow index > 4 mm) can be free of disease and some patients with thin melanoma (Breslow index < 1 mm) can die of their disease.

Current therapies have limited effectiveness, and surgery remains the mainstay of the treatment. Better treatments are certainly needed: the treatment results of advanced melanoma patients yields poor or even absence of survival benefit. Only a few of them will get a benefit from their systemic treatment: survival of AJCC stage IV melanoma patients with visceral/brain metastases can be estimated to 6–9 months. These poor therapeutic responses may be, at least in part, due to inadequate treatment or inclusion of patients in therapeutic protocols, linked to inappropriate staging.

In the past, the only prognostic factor used in melanoma patients was limited to histology (tumor thickness) and the localization of the primary tumor. These parameters remain important but have been further complemented by many clinical, pathological, and biological prognostic factors, particularly in advanced melanoma patients. Recently the use of serum markers, isolated or combined, has been suggested in order to refine the prognosis of a patient, to ensure adequate followup, and to predict the possible benefit from a therapy. Several melanoma-specific or non specific biomarkers can be found in the serum of advanced patients, and in most cases, these markers are directly correlated with tumor burden. Among all these biomarkers, S100B emerges as a protein with an independent prognostic value in advanced melanoma, more specific and sensitive than LDH, as illustrated by some studies, but not yet ideal.

Because biomarkers are a useful way to understand the biological diversity of melanoma, new biomarkers should be defined and further investigations should be carried out.

This biomarker research is important since it could improve patient monitoring, early detection, and treatment of secondary lesions and open new perspectives for targeted therapies. The multiple molecular modifications underlying melanoma progression are currently being intensely investigated. Nowadays there is a wealth of data generated by proteomic and genomic approaches, which is growing daily. These techniques allow the determination of molecular profiling of individual tumors and the study of expression of thousands of genes in order to isolate genes or family of genes of interest. It is important to recall that because of posttranslational modifications an activated gene does not mean a bioactive protein. These techniques give the possibility of classifying melanomas based on their complex biological diversity, and this will have surely a direct impact on the definition of new biomarkers and on their large scale studies.

## 4. Conclusions

In melanoma patients, still more than other diseases, there is a need for careful followup, and the question arises whether biomarkers can be useful in daily practice. So far serum tumor markers, specific for melanoma, have not routinely been used. Despite the fact that LDH is the only serum marker which have been included in the AJCC classification, we dare to think that S100B appears to be a promising protein which offers a reliable prognostic value in AJCC stage III and IV patients.

To a lesser extent because of ill-defined or poorer sensitivity/specificity, CRP, MIA, and Gal-3 can also be considered as interesting biomarkers. LDH and CRP maintain their important place in this field because of their easy availability. Other molecules such as melanin metabolites, cytokines, metalloproteinase, and adhesion proteins might be useful, but in any case, their clinical significance should be compared in prospective trials to other melanoma markers described.

Clinical research should probably now focus on combination of these molecules and distinguish their prognostic and predictive value.

Proteomic pattern study and genomic research will surely yield evidence in the next decade leading to more well-defined serum indicators of melanoma progression that can be used for early diagnosis and/or improved and tailored cancer therapy.

## Figures and Tables

**Table 1:** Melanoma-associated antigens (adapted from Visser et al. [11]).

Antigen	HLA restriction
*Oncospermatogonal antigens*	

MAGE-A1	A*01, A*03, A*24, A*28, B*3701, B*53, Cw*0201, Cw*0301, Cw*1601
MAGE-A2	A*0201, B*3701
MAGE-A3	A*01, A*02, A*2402, B*3701, B*44, DR*11
MAGE-4	A*0201
MAGE-A6	A*3402, B*3701
MAGE-A10	A*0201
MAGE-A12	A*0201
MAGE-B1	A*0201
MAGE-B2	A*0201
BAGE	Cw*1601
GAGE-1	Cw*6
LAGE-1	A*0201
PRAME	A*24
NY-ESO-1	A*02, A*31
DAM-6	A*02

*Melanocytic differentiation antigens*	

Tyrosinase	A*01, A*0201, A*2402, B*44, DR*β*1*0401
MART-1/Melan-A	A*0201, A*02, B*4501
Gp100	A*0201, A*03, A*0301, A*1101, A*2402, C*0802, DR*β*1*0401
TRP-1	A*31
TRP-2	A*31, A*33, A*0201, C*0802, A*68011
MC1R	A*0201

*Mutated antigens*	

MUM-1	B*44
CDK4	A*02
B-catenin	A*24
P15	A*24
GnT-V	A*02
TPI	DR*β*1*0101
Annexin II	DR*β*1*0401
CDC27	DR*β*1*0401

*Oncogene-derived antigens*	

HER2/Neu	A*0201
